# Description of *Nilgiriuspygoprominulus* sp. n. (Opiliones, Assamiidae, Trionyxellinae) from China, with notes on its sexual dimorphism

**DOI:** 10.3897/BDJ.11.e102954

**Published:** 2023-06-01

**Authors:** Jingjing Zhao, Zien Cheng, Chao Zhang

**Affiliations:** 1 Key Laboratory of Zoological Systematics and Application of Hebei Province, Institute of Life Science and Green Development, College of Life Sciences, Hebei University, Baoding, China Key Laboratory of Zoological Systematics and Application of Hebei Province, Institute of Life Science and Green Development, College of Life Sciences, Hebei University Baoding China; 2 International College, Hebei University, Baoding, China International College, Hebei University Baoding China

**Keywords:** Arachnida, harvestmen, new species, taxonomy, Indomalayan realm

## Abstract

**Background:**

A new species of *Nilgirius*, *N.pygoprominulus* sp. n. (male and female) in the family Assamiidae from Yunnan Province, China, is described and illustrated herein. Sexual size dimorphism (male larger than female) is inconsistent with most assamiids. Other sexually dimorphic features (body shape, leg IV and pseudonychium) are reported.

**New information:**

*Nilgiriuspygoprominulus* sp. n. is described as a new species of Trionyxellinae. Information about sexual dimorphism of the species is reported.

## Introduction

Assamiidae Sørensen, 1884 is one of the largest families of Opiliones, consisting of more than 450 described species (Kury et al. 2022b). They are widely distributed in the Afrotropics, Australasia and the Indian subcontinent, with the greatest diversity of genera and species found in sub-Saharan Africa. The classification of Assamiidae has gone through several stages, from three subfamilies (Assamiinae Roewer, 1912, Dampetrinae Sørensen, 1886 and Trionyxellinae Roewer, 1912) to 17 subfamilies by Roewer (1912) and Roewer (1935). Martens (2022) proposed a new subfamily, Filopalpinae Martens, 2022, which includes five new species. Thus, currently, Assamiidae comprises 18 subfamilies.

Assamiids exhibit a wide range of morphological diversity. Some species are completely blind, such as Irumuinae Kauri, 1985, while others have a hypertrophied fourth leg coxa and scutum in males (*Paktongius* Suzuki, 1969 and *Mysorea* Roewer, 1935). The body size of these arachnids can range from 2 to 8 mm. The carapace of the species has a row of five sharp spines on the anterior margin and the pedipalpi are flattened and crossed in front of the body. Recently, Palmieri et al. (2023) conducted a molecular phylogeny of Assamiidae using a ten-locus Sanger dataset. They sampled key taxa from both the Afrotropics and Australasia and recovered the Assamiidae as a monophyletic group, including Trionyxellidae. However, the sister group of Assamiidae remains uncertain.

**Figure 1.**
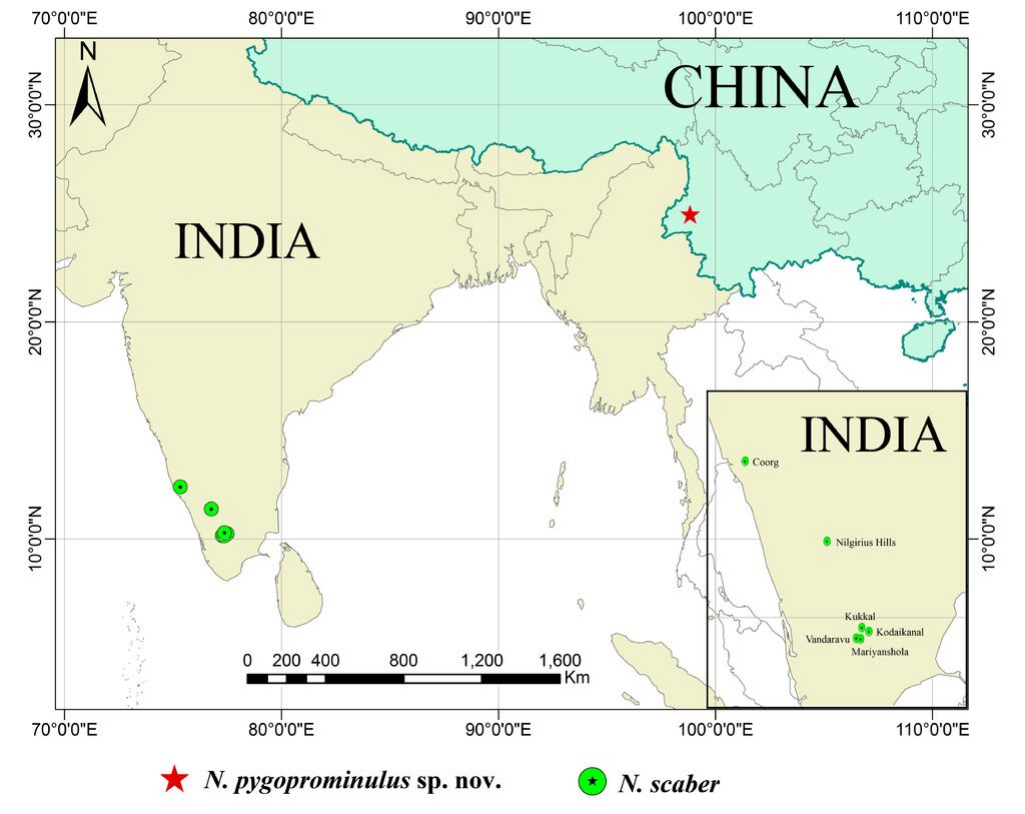
Distribution map of the genus *Nilgirius*.

The genus *Nilgirius* Roewer, 1915 (Trionyxellinae) is considered monotypic and is known only from a single specimen of the nominate species *N.scaber* Roewer, 1915. This specimen was collected from the region of Nilgiri Hills, located in the south-eastern State of Tamil Nadu, India (Roewer 1915). One of the distinguishing characteristics of this species is its peculiar feature of a pair of spines on the interocular mound. As additional specimens were collected from various regions in India, Roewer (1923), Roewer (1929), Roewer (1935) and Roewer (1939) subsequently recorded this species on multiple occasions (Fig. 1).

In the past two decades, taxonomists have described some species of assamiids (Bauer and Prieto 2009; Santos and Prieto 2010; Zhang et al. 2010; Lotz 2011; Zhang and Zhang 2015; Martens 2022), but more attention needs to be given to the detailed descriptions of the functional morphology of male genitalia. To enhance the knowledge of the morphological features of Assamiidae, particularly Trionyxellinae, we describe and illustrate the expanded and unexpanded male genitalia of *Nilgiriuspygoprominulus* sp. n. Furthermore, the sexual dimorphism of the new species is also recognised.

## Materials and methods

The specimens were preserved in 75% ethanol, examined and drawn under a Leica M205A stereomicroscope, equipped with a drawing tube. Photographs were taken using a Leica M205A stereomicroscope, equipped with a DFC 450 CCD. Tarsal claw of leg III and leg IV were photographed using an Olympus microscope, equipped with a KUY NICE CCD camera. Individual images were compiled into a composite image using Helicon Focus (http://www.heliconsoft.com/helicon/heliconfocus.html) and edited using Adobe Photoshop CS3. The male genitalia were initially placed in hot lactic acid (40–50℃) for about 1–2 min, then transferred to distilled water; the movable parts of the glans will mostly expand within 1 min (Schwendinger and Martens 2002). The terminology of genital structures follows Martens (1986) and Macías-Ordóñez et al. (2010) and the macrosetae terminology of male genitalia follows Kury and Machado (2021). Terminology for the outline of the dorsal scutum follows Kury and Medrano (2016). Type specimens of the new species are deposited in the Museum of Hebei University, Baoding, China (MHBU). All measurements are given in mm.

## Taxon treatments

### Nilgirius

Roewer, 1915

55446031-3F58-5635-B43B-CB168A6772B7

Nilgirius scaber Roewer, 1915 - type species

#### Diagnosis

Medium-sized assamiids (3.00–5.00) with the spine on the interocular mound. Carapace, scutal areas and tergites unarmed, except for some acuminate tubercles. Basichelicerite of chelicerae armed with tubercles. Pedipalpal femur distally with one setiferous tubercle on medial side. Femora of legs III–IV slightly curved and tarsi III–IV each with a pseudonychium and two simple claws. Coxa IV extremely enlarged in male and the distance from genital operculum to the distal coxa IV is about twice the distance from genital operculum to anterior margin of coxa I. Distitarsus I two-jointed. Distitarsus II three-jointed, rarely four-jointed. Tarsal formula (I–IV): 5(2)/9–11(3)/6–7(3)/7(3). Spiracles concealed.

#### Distribution

China (Baoshan City), India (Nilgiris Hills, Kodaikanal, Vandaravu, Mariyanshola Forest, Kukkal, Coorg).

##### Included species (two species)

*Nilgiriusscaber* Roewer, 1915 and *Nilgiriuspygoprominulus* sp. n.

#### Notes

Only one species was included in the genus before the description of this new species.

### Nilgirius pygoprominulus

Zhao & Zhang sp. n.

D67EACBE-5901-5806-9F8A-92391C89F968

EC045E53-03D3-4326-A78C-13ABE89C6D02

#### Materials

- **Type status:** Holotype. **Occurrence:** individualID: MHBU-Opi-17ZC1101m; individualCount: 1; sex: male; lifeStage: adult; occurrenceID: 5C7FD976-0913-5430-9604-D0E35898A1BD; **Taxon:** scientificName: *Nilgiriuspygoprominulus*; class: Arachnida; order: Opiliones; family: Assamiidae; **Location:** country: China; countryCode: CHN; stateProvince: Yunnan; municipality: Baoshan; locality: Lujiang Town; verbatimElevation: 164m; decimalLatitude: 24.947778; decimalLongitude: 98.832778; **Identification:** identifiedBy: Chi Jin; **Event:** samplingProtocol: by hand; year: 2017; month: 5; day: 29; **Record Level:** institutionID: the Museum of Hebei University; institutionCode: MHBU
- **Type status:** Paratype. **Occurrence:** individualID: MHBU-Opi-17ZC1101f–1103f; individualCount: 3; sex: female; lifeStage: adult; occurrenceID: 9C77CD26-48D7-57B1-8571-3E7A693E74A8; **Taxon:** scientificName: *Nilgiriuspygoprominulus*; class: Arachnida; order: Opiliones; family: Assamiidae; **Location:** country: China; countryCode: CHN; stateProvince: Yunnan Province; municipality: Baoshan; locality: Lujiang Town; verbatimElevation: 164m; decimalLatitude: 24.947778; decimalLongitude: 98.832778; **Identification:** identifiedBy: Chi Jin; **Event:** samplingProtocol: by hand; year: 2017; month: 5; day: 29; **Record Level:** institutionID: the Museum of Hebei University; institutionCode: MHBU

#### Description

**Figure 2.**
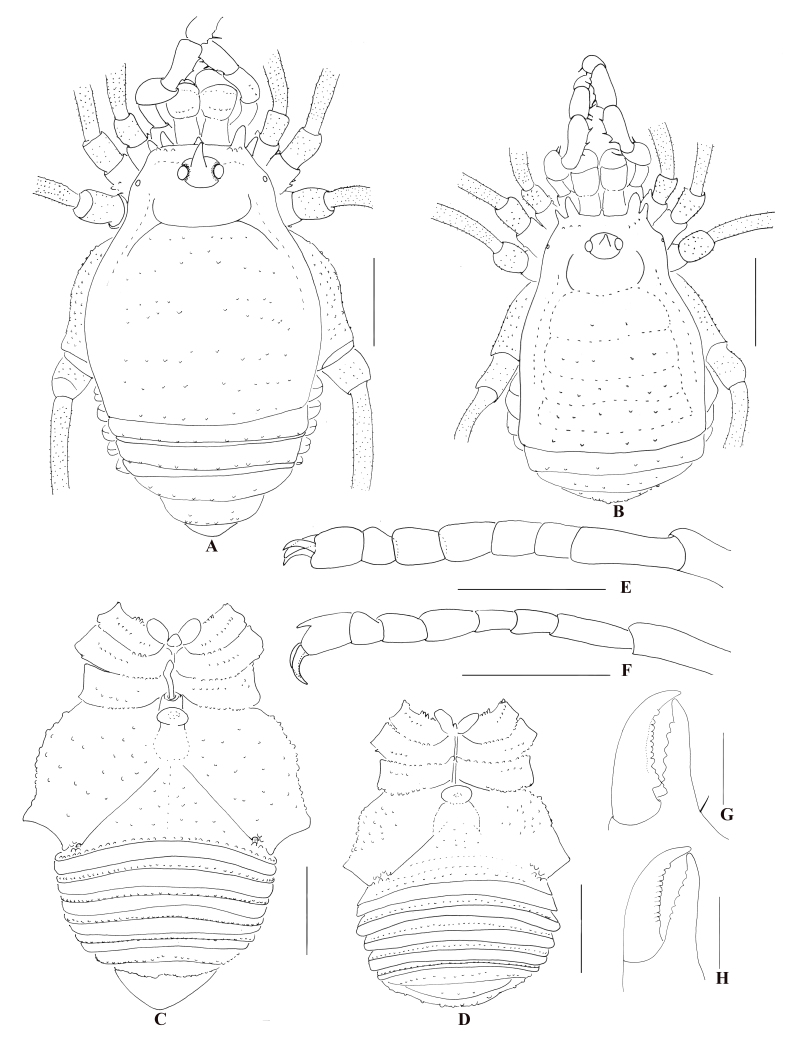
*Nilgiriuspygoprominulus* sp. n., male holotype and female paratype (MHBU-Opi-17ZC1101f). **A** Male body, dorsal view; **B** Female body, dorsal view; **C** Male body, ventral view; **D** Female body, ventral view; **E** Right tarsal claw IV of male, lateral view; **F** Right tarsal claw IV of female, lateral view; **G** Cheliceral fingers of male, frontal view; **H** Cheliceral fingers of female, frontal view. Scale bars: 1 mm (**A–D**); 0.5 mm (**E**, **F**); 0.25 mm (**G**, **H**).

**Figure 3.**
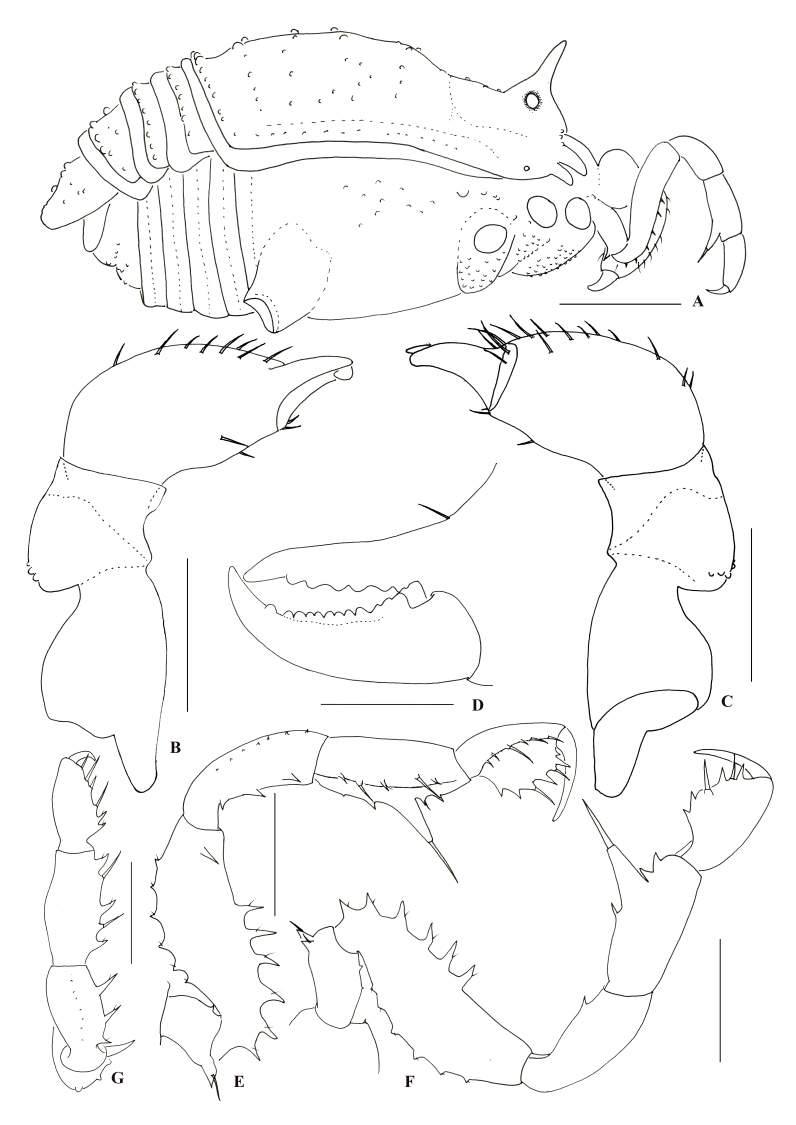
*Nilgiriuspygoprominulus* sp. n., male holotype. **A** Male body, lateral view; **B** Left chelicera of male, ental view; **C** Same, ectal view; **D** Left cheliceral fingers, frontal view; **E** Left pedipalp of male, ental view; **F** Left pedipalp of male, ectal view; **G** Left pedipalp of male, dorsal view. Scale bars: 1 mm (**A**); 0.5 mm (**B**, **C**, **E–G**); 0.25 mm (**D**).

**Male holotype**. Habitus as presented in Fig. 2A, C, Fig. 3A and Fig. 5A–C. Colouration in ethanol: entire body dorsally rusty yellow with dark brown patches; median area of prosoma with dark brown reticulations before and behind the interocular mound; both lateral ridges of scutum with dark brown stripes; dorsal scutal areas I–V and free tergites dark brown with transverse paler interspaces. Coxae with light brown patches on edges and surface. Chelicerae, pedipalpi and legs reticulated with light to dark brown.

**Dorsum** (Fig. 2A; Fig. 5A). Dorsal scutum (DS) pyriform, with the widest portion at scutal area II, as a typical beta shape（Fig. 2A; Fig. 5A). Mid-bulge symmetrical, centred around mid-DS, covering two-thirds of scutum length. Carapace narrower than mid-bulge, subparallel, widening posteriorly. Posterior border of scutum moderately convex, narrower than mid-bulge. Anterior margin of carapace with two spines at the lateral portion and a single median spine, all similar spines directed horizontally, the middle one is the smallest. Interocular mound oval and armed with a spine median longer than the height of interocular mound (Fig. 3A, lateral view). Scutal areas I–V each with a row of acuminate tubercles and a longitudinal row of similar tubercles on the lateral margins. Free tergites and anal operculum also armed with a row of tubercles that are more slender and dapper than scutal areas I–V.

**Venter** (Fig. 2C; Fig. 5C). Genital operculum and free sternites with hair-tipped granules. Spiracles concealed. Coxa I–IV with a row of marginal tubercles at the border and tuberculated on the ventral surface. Coxa IV enlarged.

**Chelicerae** (Fig. 2G; Fig. 3B–D). Basichelicerite elongate, with distinct bulla, no prominent armaments, except five small tubercles. Movable finger with twelve mound-shaped teeth; fixed finger with six teeth.

**Pedipalpi** (Fig. 3E and F). Trochanter ventrally with an enlarged setiferous tubercle and a small one. Femur dorsally with a row of six small setiferous tubercles, ventrally with a row of six setiferous tubercles; meso-apically with one setiferous tubercle. Patella ventro-mesally with three and ventro-ectally with one tubercle. Tibia ventro-mesally with five and ventro-ectally with an enlarged tubercle and three small tubercles. Tarsus ventro-mesally and ventro-ectally each with six setiferous tubercles and tarsal claw slightly curved, shorter than tarsus.

**Legs** (Fig. 2E; Fig. 6A and B). All segments rounded in cross section, with scattered small tubercles. Femora III–IV slightly curved, distitarsi I with two tarsomeres and distitarsi II–IV with three tarsomeres. Tarsi III–IV each with a pseudonychium (Fig. 2E; Fig. 6A and B) and two bare claws. Tarsal formula (I–IV): 5(2)/10(3)/6(3)/7(3).

**Figure 4.**
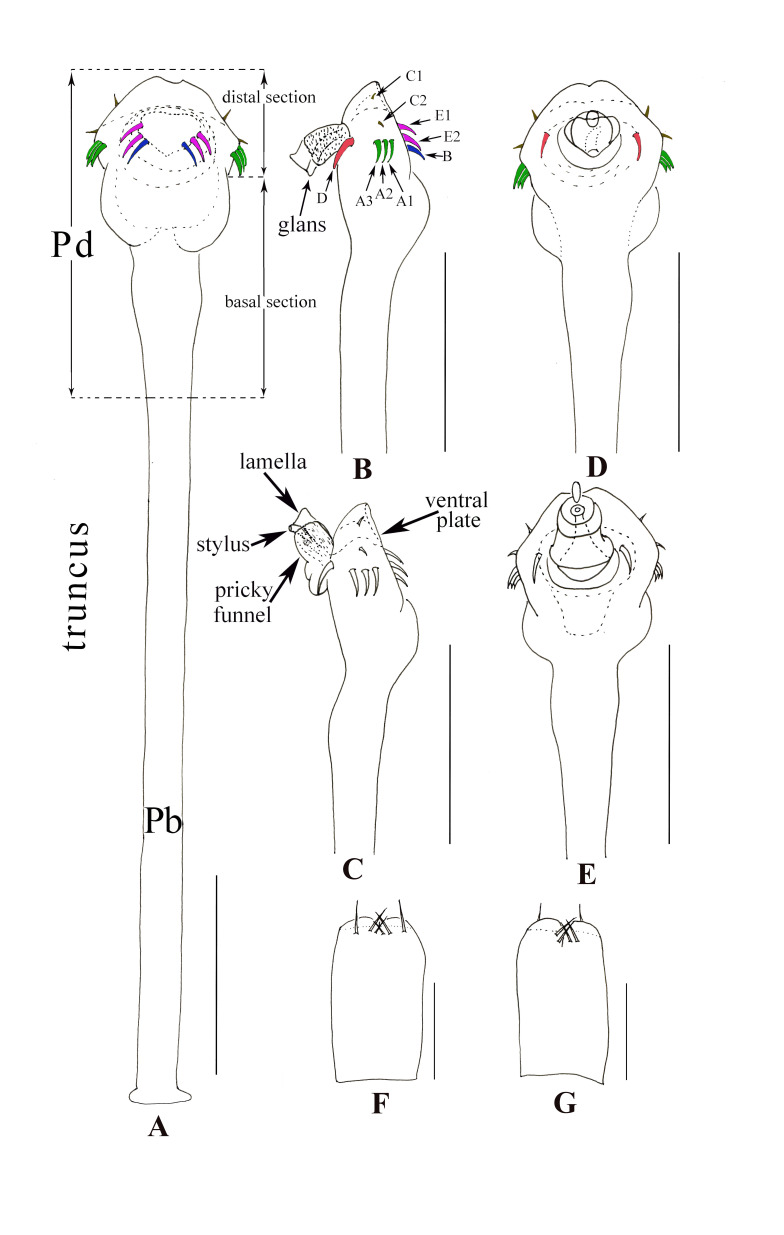
*Nilgiriuspygoprominulus* sp. n., genitalia of male holotype and female paratype (MHBU-Opi-17ZC1101f). **A** Penis, ventral view; **B** Distal part of penis, lateral view; **C** Expanded, same; **D** Distal part of penis, dorsal view; **E** Expanded, same; **F** Ovipositor, dorsal view; **G** Same, ventral view. Scale bars: 0.25 mm.

**Penis** (Fig. 4A–E). Truncus (pars basalis) without muscle and tendon system, slender, sides nearly parallel, then slightly enlarged (Fig. 4A, ventral view) and curved (Fig. 4B and C, lateral view) towards distal end. Distal portion of penis (pars distalis) markedly enlarged: ventral plate nearly triangle and frontal rim with median crevice (Fig. 4A, ventral view), convex in dorsal view and concave in ventral view (Fig. 4B and C, lateral view); basal section mainly with two rounded lobes, convex in ventral view (Fig. 4A and D, ventral and dorsal view). Glans composed of two-thirds of prickly funnel near the base and one-third of stylus and lamella (Fig. 4B and C, lateral view). The distal portion of truncus (pars distalis) with 18 macrosetae distributed symmetrically. Five pairs of macrosetae inserted in both sides of ventral plate, the distal C1–C2 form a vertical line comparatively with the transversal A1–A3 proximally. Three pairs of macrosetae ventrally close to the centre of distal section (Fig. 4A), one pair of proximal B proximally and two pairs of E1–E2 distally. One pair of D near the basal glans.

**Figure 5.**
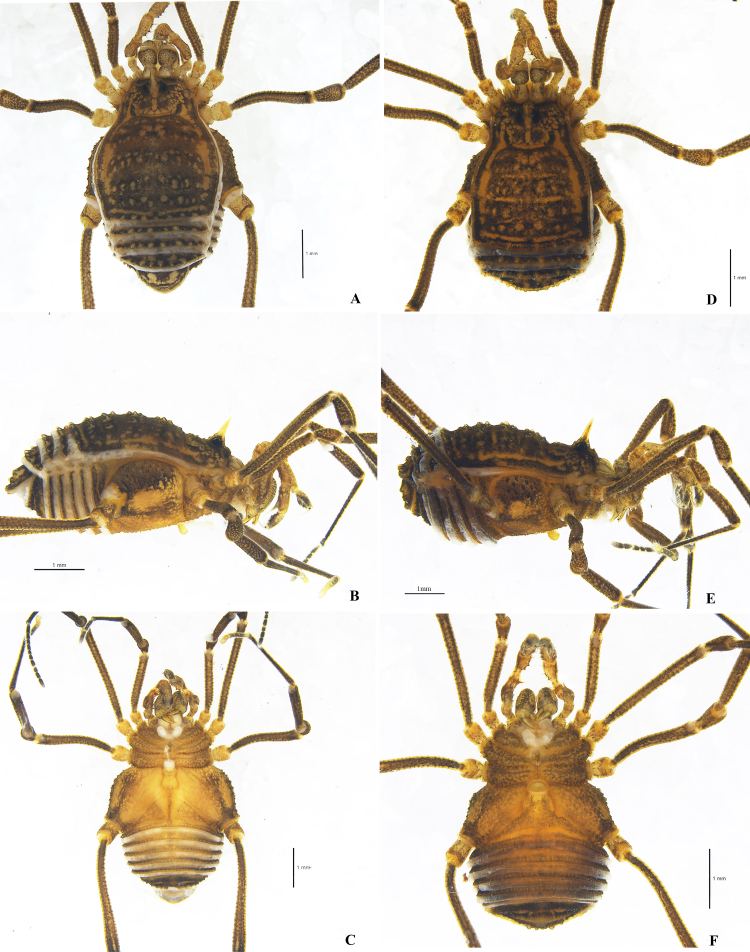
Photographs of male (**A–C**, holotype) and female (**D–F**, paratype, MHBU-Opi-17ZC1101f) of *Nilgiriuspygoprominulus* sp. n.. **A** Male body and parts of appendages, dorsal view; **B** Same, lateral view; **C** Same, ventral view; **D** Female body and parts of appendages, dorsal view; **E** Same, lateral view; **F** Same, ventral view. Scale bars: 1 mm.

**Figure 6.**
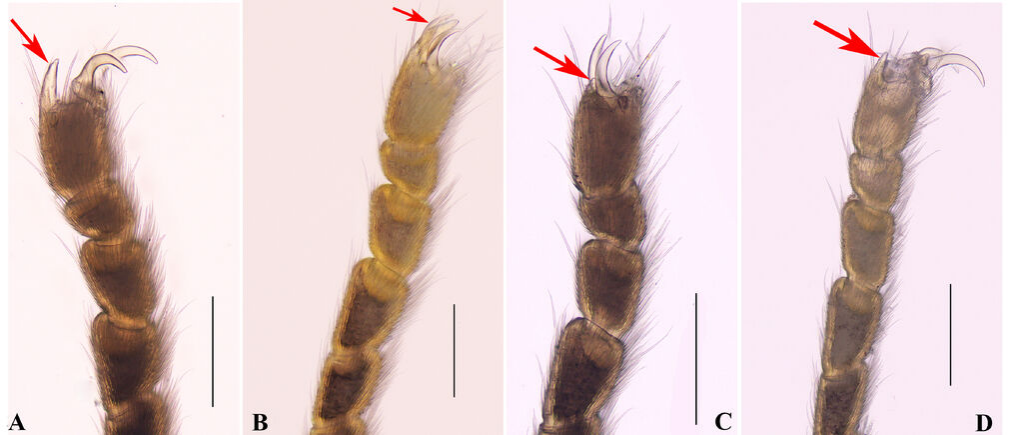
Photographs of pseudonychium of leg III–IV in male holotype and female paratype (MHBU-Opi-17ZC1101f). **A–B** Pseudonychium of leg III–IV in male (holotype); **C–D** Pseudonychium of leg III–IV in female paratype (MHBU-Opi-17ZC1101f). Scale bars: 0.25 mm.

**Female** (Fig. 2B, D, F and H; Fig. 4F and G; Fig. 5D–F; Fig. 6C and D). Similar in colouration to male. Body size smaller than male. Scutum approximately trapezoidal. Legs and pedipalpi much shorter and thinner than those of male (Tables 1, 2). Pseudonychium of legs III–IV in female reduced compared to that of male (Fig. 2E and F; Fig. 6). Tarsal formula (I–IV): 5(2)/9(3)/6(3)/7(3). Movable finger of chelicera with eleven round teeth in female.

**Ovipositor** (Fig. 4F and G). Short. Ventral surface with four setae and dorsal surface with six setae.

**Sexual dimorphism**. (1) body of male much larger than that of females; (2) dorsal scutum slightly piriform in male and approximately trapezoidal in females; (3) coxa of leg IV and pseudonychia of leg III–IV enlarged in male; (4) male leg IV much longer than female’s; (5) teeth on cheliceral finger in male a bit more robust than those of females.

**Measurements**. Male holotype (female paratype): Body 4.66 (3.35) long, 2.74 (2.09) wide at the widest portion. Scutum 3.65 (2.49) long. Interocular mound 0.40 (0.39）long, 0.26 (0.25) wide, 0.19 (0.18) high, 0.18 (0.15) far from the anterior border of the scutum. Pedipalpal claw 0.32 (0.26) long. Measurements of left pedipalpus and legs as in Tables 1, 2.

#### Diagnosis

The distal portion of male genital truncus (pars distalis) markedly enlarged and with two rounded lobes (Fig. 4A). Penial macroseate A clustered transversally on the lateral vetral plate (Fig. 4B). Dorsal scutum of male outline of type beta (Fig. 2A; Fig. 5A). Scutal areas I–V with scattered tubercles, free tergites each with transverse rows of acuminate tubercles. Interocular mound with one single large spine (Fig. 3A; Fig. 5B and E). Pedipalpal femur meso-apically with a setiferous tubercle and tibia ventro-ectally with an enlarged tubercle and three small tubercles (Fig. 3E and F).

#### Etymology

The specific epithet combines the Greek word pyge, meaning "rump, buttocks", plus the Latin adjective prominulus, meaning “tubercle" or "spine”. Pyge refers to the shape of penis and prominulus refers to the tubercle of the dorsal scutumn and legs of the new species.

#### Distribution

Known only from the type locality.

## Discussion

**Taxonomy**

The limited ability of Opiliones species to disperse and their narrow distribution are the primary reasons for studying their biogeography. *Nilgiriusscaber* has been mentioned repeatedly by Roewer (1915, 1923, 1929, 1935, 1939) and all specimens have been found in southern India (Nilgiris Hills, Kodaikanal, Vandaravu, Mariyanshola Forest, Kukkal, Coorg). By contrast, *N.pygoprominulus* sp. n. was collected from southern China (Baoshan City, Yunnan Province). The two localities are nearly 2600 km apart (see Fig. 1), indicating a disjunction between the two species. Additionally, the altitude of the habitats for both species is different, with *N.pygoprominulus* sp. n. being recorded at 164 m, while some specimens of *N.scaber* were found at altitudes of 1800–2350 m (Roewer 1929: 621). The disjunction and the differences in habitat suggest the possibility of two different evolutionary lineages. Note that Palmieri et al. (2023) found a sister-group relationship in a clade composed of "Indian Region" and "Thai-Malay Peninsula" which is replicated here in the species of *Nilgirius*, considering that Yunnan is more Thai-Malay than "Eastern Palearctic".

*Nilgiriuspygoprominulus* sp. n. has distinct external morphological characteristics compared to *N.scaber*. The interocular mound of the new species is armed with a single spine, whereas *N.scaber* has two spines. Additionally, the anterior margin of the carapace of *N.pygoprominulus* sp. n. has two spines at the lateral portion, whereas *N.scaber* has an extra small spine between the two spines. Furthermore, *N.pygoprominulus* sp. n. has five tubercles on the dorsal basichelicerite instead of being smooth like *N.scaber*.

As the genus *Nilgirius* was erected in 1915, Roewer (1915) placed it in the subfamily Trionyxellinae Roewer, 1912. There are still inconsistent opinions on whether the ‘Trionyxellinae’ is a family or a subfamily (Kury 2018; Palmieri et al. 2023). We tentatively place Trionyxellinae as a subfamily of Assamiidae *s.l.*

Within the subcontinental Trionyxellinae, there are eight genera with very close geographical locations. Five genera (*Balnissa* Roewer, 1935, *Brysma* Roewer, 1935, *Calloristus* Roewer, 1935, *Trionychiperna* Roewer, 1929 and *Nilgirius*) were recorded from the Deccan (India). Three genera (*Kandyca* Roewer, 1915, *Nuwaria* Roewer, 1915 and *Trionyxella* Roewer, 1912) in Sri Lanka. Additionally, *Nuwaria* possessed the most similar external morphology with *Nilgirius* according to the limited original description. There is a supposition that the eight genera may be the most likely to converge into a molecular clade.

Obviously, our understanding of the Assamiidae *s.l.* in China is still in its early stages. Currently, only one species, *Nilgiriuspygoprominulus* sp. n., has been identified. While there is a lack of information on the genital morphology of most assamiids, we can classify this new species as belonging to the group of ‘the Sri Lankan/Indian pseudonychiate genera’ (Kury 2007). This classification is based on the presence of a pseudonychium on tarsi III–IV of the legs, which is a typical morphological characteristic of these legs. The presence of the pseudonychium in the legs III–IV may be an ancestral trait of the group.

**Sexual dimorphism**

Sexual dimorphism appears in various forms in Opiliones, including differences in body size, length and armature of chelicerae, legs and pedipalpi and glandular openings on legs, pedipalpi and chelicerae (e.g. Dasilva and Kury 2007; Buzatto and Machado 2008; Willemart et al. 2009; Willemart et al. 2010; Buzatto et al. 2011; Zatz et al. 2011; Painting et al. 2015; Solano-Brenes et al. 2018). The typical sexual dimorphism of assamiids is reinforced in the appendages of male, for example, enlarged leg II and leg IV, swollen cheliceral hand (Sharma et al. 2011; Kury et al. 2022a) and filiform pedipalpi in Filopalpinae (Martens 2022).

*Nilgiriuspygoprominulus* sp. n. also possesses enlarged appendages in the male (coxa IV and leg IV). However, there is no difference in sexual dimorphism in the pedipalpi and chelicerae, except for variations in the number of teeth on the cheliceral fingers. The most conspicuous sexual dimorphism is that the male body is larger than the female’s, instead of the female being larger than the male. Additionally, the pseudonychium of legs III–IV in the male are stronger than those of females. From the morphological charateristics, especially the larger body and reinforced leg IV in male, we tentatively suppose that it is more likely to help *Nilgiriuspygoprominulus* sp. n. win in male-male competition and are more likely to be preferred by females.

A great variety of forms of sexual dimorphism in Opiliones suggest that sexual selection, which acts by intersexual selection (female choice) and intrasexual selection (male-male contest), may have played an important role in their evolution (Buzatto et al. 2014).

Sexual dimorphism in the leg armature appears in diverse forms in Opiliones and is associated with functional meaning. A study focusing on *Neosadocusbufo* (Giltay, 1928) showed that the leg armature was used as a weapon in contests between males (suggested fight for specific sites and individual females; Willemart et al. 2009). Territorial behaviour in males may be associated with a greater development of proximal musculature of leg IV, causing their coxa to be largely developed.

## Supplementary Material

XML Treatment for Nilgirius

XML Treatment for Nilgirius pygoprominulus

## Figures and Tables

**Table 1.** *Nilgiriuspygoprominulus* sp. n. Measurements of the pedipalpus and legs of the male holotype, length/depth.

	Trochanter	Femur	Patella	Tibia	Metatarsus	Tarsus	Total
Pedipalpus	0.44/0.21	0.96/0.25	0.48/0.23	0.62/0.27		0.37/0.23	2.87
Leg I	0.24/0.22	1.50/0.22	0.53/0.24	1.10/0.21	1.52/0.10	0.89/0.09	5.78
Leg II	0.40/0.31	2.52/0.20	0.83/0.28	2.11/0.18	2.41/0.09	1.85/0.10	10.12
Leg III	0.42/0.34	1.98/0.26	0.62/0.33	1.31/0.25	1.93/0.13	1.02/0.12	7.28
Leg IV	0.45/0.40	2.78/0.33	1.06/0.44	1.88/0.48	3.31/0.17	1.28/0.12	10.76

**Table 2.** *Nilgiriuspygoprominulus* sp. n. Measurements of the pedipalpus and legs of the female paratype, length/depth.

	Trochanter	Femur	Patella	Tibia	Metatarsus	Tarsus	Total
Pedipalpus	0.36/0.20	0.88/0.25	0.40/0.21	0.54/0.26		0.41/0.17	2.59
Leg I	0.35/0.25	1.10/0.20	0.56/0.22	0.89/0.19	1.35/0.08	0.80/0.08	5.05
Leg II	0.40/0.27	2.08/0.17	0.61/0.25	1.73/0.18	2.06/0.07	1.60/0.09	8.48
Leg III	0.42/0.30	1.62/0.21	0.59/0.28	1.04/0.23	1.82/0.11	0.74/0.10	6.23
Leg IV	0.48/0.30	2.22/0.17	0.63/0.30	1.45/0.21	2.88/0.09	1.16/0.10	8.82

## References

[B9145194] Bauer Christian, Prieto Carlos (2009). Three new Assamiidae (Arachnida: Opiliones) from Cameroon, with a redescription of *Chilonrobustus* and comments on related species. Zootaxa.

[B9145203] Buzatto Bruno Alves, Machado Glauco (2008). Resource defense polygyny shifts to female defense polygyny over the course of the reproductive season of a neotropical harvestman. Behavioral Ecology and Sociobiology.

[B9145222] Buzatto Bruno Alves, Requena Gustavo S, Lourenço Rafael S, Munguía-Steyer Roberto, Machado Glauco (2011). Conditional male dimorphism and alternative reproductive tactics in Neotropical arachnid (Opiliones). Evolutionary Ecology.

[B9145232] Buzatto Bruno Alves, Tomkins Joseph L, Simmons Leigh W, Machado Glauco (2014). Correlated evolution of sexual dimorphism and male dimorphism in a clade of Neotropical harvestmen. Evolution.

[B9145241] Dasilva MARCIO B, Kury ADRIANO B (2007). A remarkable new species of *Multumbo* showing sexual dimorphism, with the transfer of *Multumbo* and *Piassagera* to the Hernandariinae (Opiliones, Gonyleptidae). Zootaxa.

[B9746420] Kury Adriano Brilhante, Pinto-Da-Rocha Ricardo, Machado Glauco, Giribet Gonzalo (2007). Harvestmen: The biology of Opiliones.

[B9145296] Kury A B, Medrano M (2016). Review of terminology for the outline of dorsal scutum in Laniatores (Arachnida, Opiliones). Zootaxa.

[B9145277] Kury A B (2018). Familial nomina in harvestmen (Arachnida, Opiliones). Binomina.

[B9746285] Kury Adriano Brilhante, Machado Glauco (2021). The genus *Eurytromma* from Sri Lanka: the homology of penial macrosetae in Podoctidae matches the gonyleptoid AE11 pattern (Opiliones: Laniatores: Epedanoidea). The Journal of Arachnology.

[B9145286] Kury A B, Bernabé T N, deÁzara L N, Araújo D, Benedetti A R (2022). Phylogeny of the clade K92 (Opiliones, Laniatores, Gonyleptidae) with description of a new subfamily and discussion on the evolution of caelopygine facies and sexual dimorphism. Zoologischer Anzeiger.

[B9145313] Kury A B, Mendes A C, Cardoso L, Kury M S, Granado Ade A, Giribet G, Cruz-López J A, Longhorn S J, Medrano M, Kury I S, Souza-Kury M A World Catalogue of Opiliones. WCO-Lite version 2.5.0.. https://wcolite.com/.

[B9145337] Lotz L N (2011). Three new harvestmen species from southern Africa (Arachnida: Opiliones: Caddidae, Neopilionidae, Assamiidae). Journal of Afrotropical Zoology.

[B9145959] Macías-Ordóñez R, Machado G, Pérez-González A, Shultz J W, Leonard J, Córdoba-Aguilar A (2010). The evolution of primary sexual characters in animals.

[B9145394] Martens J (1986). Opiliones aus dem Nepal-Himalaya. III. Oncopodidae, Phalangodidae, Assamiidae (Arachnida). Senckenbergiana biologica.

[B9145376] Martens Jochen (2022). From the Ethiopian Bale Mountains hotspot—Filopalpinae subfam. nov., a new taxon of Laniatorean harvestmen based on external and genital morphology (Arachnida, Opiliones, Assamiidae). Zootaxa.

[B9145412] Painting C J, Probert A F, Townsend D J, Holwell G J (2015). Multiple exaggerated weapon morphs: a novel form of male polymorphism in harvestmen. Scientific Reports.

[B9145421] Palmieri L, Giribet G, Sharma P P (2023). Too early for the ferry: The biogeographic history of the Assamiidae of southeast Asia (Chelicerata: Opiliones, Laniatores). Molecular Phylogenetics and Evolution.

[B9145741] Roewer C F (1912). Die Familien der Assamiiden und Phalangodiden der Opiliones-Laniatores. (= Assamiden, Dampetriden, Phalangodiden, Epedaniden, Biantiden, Zalmoxiden, Samoiden, Palpipediden anderer Autoren). Archiv für Naturgeschichte, Berlin, Abt. A, Original-Arbeiten.

[B9746358] Roewer Carl Friedrich (1915). 106 neue Opilioniden. Archiv für Naturgeschichte.

[B9164721] Roewer C F (1923). Die Weberknechte der Erde. Systematische Bearbeitung der bisher bekannten Opiliones..

[B9746376] Roewer Carl Friedrich (1929). Süd-indische Skorpione, Chelonethi und Opilioniden. Revue suisse de zoologie, Genève.

[B9167745] Roewer C F (1935). Alte und neue Assamiidae. Weitere Weberknechte VIII (8. Ergänzung der "Weberknechte der Erde" 1923). Veröffentlichungen aus dem Deutschen Kolonial-und Übersee-Museum in Bremen, Bremen.

[B9746385] Roewer Carl Friedrich (1939). On a collection of Indian Opiliones of the Government Museum of Madras. Records of the Indian Museum.

[B9145776] Santos R, Prieto C (2010). Los Assamiidae (Opiliones: Assamiidae) de Río Muni (Guinea Ecuatorial), con la descripción de ochonuevase species. Revista De Biologia Tropical.

[B9145785] Schwendinger P J, Martens J (2002). A taxonomic revision of the family Oncopodidae III: Further new species of *Gnomulus* Thorell (Opiliones, Laniatores). Revue Suisse de Zoologie.

[B9145794] Sharma P P, Prieto C E, Giribet G (2011). A new family of Laniatores (Arachnida: Opiliones) from the Afrotropics. Invertebrate Systematics.

[B9145813] Solano-Brenes D, García-Hernández S, Machado G (2018). All the better to bite you with! Striking intrasexual differences in cheliceral size define two male morphs in an Amazonian arachnid. Biological Journal of the Linnean Society.

[B9145890] Willemart R H, Osses F, Chelini M C, Macias-Ordonez R, Machado G (2009). Sexually dimorphic legs in a Neotropical harvestman (Arachnida, Opiliones): ornament or weapon?. Behavioural Processes.

[B9145914] Willemart R H, Pérez-Gonzalez A, Farine J-P, Gnaspini P (2010). Sexually dimorphic tegumental gland openings in Laniatores (Arachnida, Opiliones), with new data on 23 species. Journal of Morphology.

[B9145923] Zatz C, Werneck R M, Macias-Ordoñez R, Machado G (2011). Alternative mating tactics in dimorphic males of the harvestman Longiperna concolor (Arachnida: Opiliones). Behavioral Ecology and Sociobiology.

[B9145941] Zhang C, MacDermott J, Zhang F (2010). First Description of the male of *Bandonaboninensis* Suzuki 1974 (Opiliones: Laniatores: Assamiidae). Acta Arachnologica.

[B9145950] Zhang C, Zhang F (2015). The assamiids harvestmen (Opiliones: Laniatores: Assamiidae) from Champasak Province, Laos. Zootaxa.

